# A real-world drug safety surveillance study from the FAERS database of hepatocellular carcinoma patients receiving durvalumab in combination with tremelimumab

**DOI:** 10.3389/fimmu.2025.1657398

**Published:** 2025-10-29

**Authors:** Yang Cheng, Mingji Zhang, Yi Yao, Mingzuo Wang, Zhong Xue, Zhaoshuo Chen, Fan Zhang

**Affiliations:** Department of Hepatobiliary Surgery, Clinical Oncology School of Fujian Medical University, Fujian Cancer Hospital, Fuzhou, Fujian, China

**Keywords:** durvalumab, tremelimumab, hepatocellular carcinoma, pharmacovigilance, FAERS, real-world

## Abstract

**Objective:**

Durvalumab plus tremelimumab has emerged as a key therapeutic option for unresectable hepatocellular carcinoma (HCC). This study aimed to meticulously monitor and identify its safety profile using real-world data from the Food and Drug Administration Adverse Event Reporting System (FAERS).

**Methods:**

Data were retrieved from the FAERS database for HCC patients who received durvalumab plus tremelimumab between the fourth quarter of 2017 and the fourth quarter of 2024. Significant adverse event (AE) signals were identified using the odds ratio (ROR), proportional reporting ratio (PRR), Bayesian confidence propagation neural network (BCPNN), and mu-item gamma Poisson shrinker (MGPS). Time-to-onset (TTO) was analyzed using Kaplan-Meier method and Weibull modeling. Independent risk factors for drug-related mortality were determined via LASSO-Cox regression, and a risk prediction model was developed to assess prognostic value.

**Results:**

Disproportionality signals were identified in 51 preferred terms (PTs) across 16 system organ classes. Notable PTs with strong signals included immune-mediated hepatic disorder, immune-mediated enterocolitis, and cytokine release syndrome. Several unexpected AEs were observed, such as thyrotoxic crisis and ulcerative colitis. Anaphylactic reaction emerged as an unexpected signal and was categorized by the European Medicines Agency as both a designated and important medical event. TTO analysis revealed that most AEs (63.21%) occurred within 30 days of administration, with a median TTO of 25 days. The occurrence of AEs was significantly influenced by age and AE type. Both exploratory LASSO-Cox regression analysis and risk prediction model preliminarily showed that immune thrombocytopenia, immune-mediated dermatitis, immune-mediated enterocolitis, immune-mediated myocarditis, multiple organ dysfunction syndrome, and myocarditis were independent risk factors for drug-related mortality.

**Conclusion:**

This pharmacovigilance study describes the safety profile of durvalumab plus tremelimumab in HCC. The findings may inform clinical monitoring strategies, though prospective studies are warranted for confirmation.

## 1 Introduction

Hepatocellular carcinoma (HCC) is the most prevalent primary liver cancer and is the third leading cause of cancer-related mortality worldwide (1). Conventional treatments offer limited benefit for patients with advanced disease. In this context, the combination of programmed death 1 (PD-1) inhibitor and cytotoxic T-lymphocyte antigen 4 (CTLA-4) inhibitor, which target distinct but complementary immune pathways, has emerged as a promising strategy for advanced HCC by enhancing endogenous antitumour immune response (2, 3).

Durvalumab, a human immunoglobulin G1 kappa (IgG1κ) monoclonal antibody, inhibits programmed death ligand 1 (PD-L1) binding to PD-1 and cluster of differentiation 80 (CD80) (4), with diarrhea, transaminase elevation, and fatigue being the most frequently reported adverse events (AEs) (5, 6). Tremelimumab, a fully human IgG2 monoclonal antibody targeting CTLA-4 interaction with CD80 and CD86 (7), is most commonly associated with rash, diarrhea, colitis, and elevated liver enzymes (8). Mechanistically, tremelimumab led to tumor-directed T-cell activation and expansion, while durvalumab further augments T-cell function and induces durable antitumor activity (3). In the phase 3 HIMALAYA trial, this complementary activity translated into a significant overall survival benefit with an overall AE profile consistent with expectations, leading to its US Food and Drug Administration (FDA) approval as a first-line treatment for unresectable HCC (9–12).

Despite its therapeutic potential, this dual ICI regimen may be associated with a distinct constellation of organ-specific inflammatory side effects or immune-related adverse events (irAEs), due to the mechanisms of immunotherapies (13). In the Asian subgroup of the HIMALAYA trial, treatment-related adverse events (TRAEs) were more frequent with durvalumab plus tremelimumab than with durvalumab alone, underscoring the need for close monitoring with this dual ICI regimen (14). Nevertheless, AEs in real-world clinical practice require systematic evaluation, particularly those that emerge with long-term exposure or may be undercharacterized in clinical trials (15).

The FDA Adverse Event Reporting System (FAERS), a spontaneous reporting database monitoring the safety of drugs and biologics, provides valuable insights into the safety profiles of approved therapies (16). Given the limited safety data on durvalumab plus tremelimumab, this study utilized the FAERS database to characterize its real-world safety profile in HCC, aiming to inform risk mitigation and optimize clinical application.

## 2 Materials and methods

### 2.1 Data source and collection

**Figure 1:**
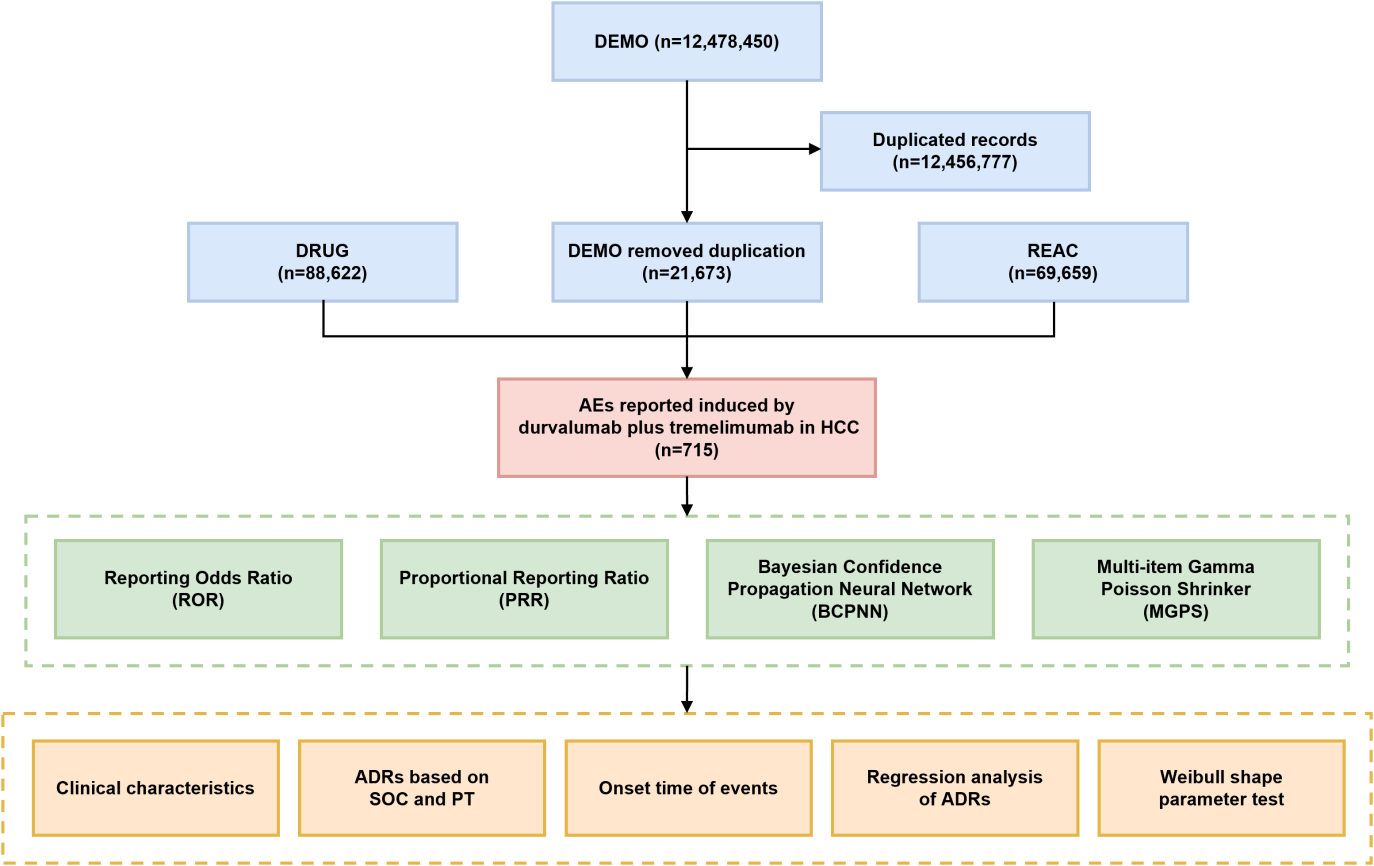
Flowchart of this study. DEMO, demographic and administrative information; DRUG, drug information; REAC, preferred terminology for adverse events; AE, adverse event; ADR, adverse drug reaction; SOC, system organ class; PT, preferred term.

This study utilized data from the FAERS, which includes seven datasets: demographic and administrative information (DEMO), drug information (DRUG), adverse drug reaction information (REAC), patient outcomes information (OUCT), reported sources (RPSR), drug therapy start dates and end dates (THER), and indications for drug administration (INDI). AE reports submitted between the fourth quarter of 2017 and the fourth quarter of 2024 were extracted, based on the marketing times of durvalumab and tremelimumab. To ensure data quality, duplicate reports were handled following FDA-recommended practices (17). A fuzzy search of the “DRUGNAME” field in the DRUG table was performed using both generic (durvalumab, tremelimumab) and trade (IMFINZI, IMJUDO) names of the drugs. The term ‘hepatocellular carcinoma’ was used to retrieve relevant reports from the INDI table based on drug indication. To identify potential AEs signals associated with the combination of durvalumab and tremelimumab, we prioritized reports where both agents were listed as primary suspect (PS) drugs (18). For data completeness, reports where the drugs were marked as secondary suspects (SS), concomitant (C), or interacting (I) were also included. AEs were coded as preferred terms (PTs) and system organ classes (SOCs) using the Medical Dictionary for Regulatory Activities (MedDRA 27.1) (19). Based on the lists laid down by the European Medicines Agency (EMA), designated medical events (DMEs) and important medical events (IMEs) are mapped to corresponding PTs to identify potentially serious and specific safety signals (20–22). Unexpected signals were identified as significant AEs not listed in the corresponding drug package inserts. A multistep process of data extraction and analysis is illustrated in **Figure 1**.

### 2.2 Signal mining and disproportionality analysis

We conducted a disproportionality analysis using four established algorithms: Reporting Odds Ratio (ROR), Proportional Reporting Ratio (PRR), Bayesian Confidence Propagation Neural Network (BCPNN), and Multi-Item Gamma Poisson Shrinker (MGPS) (23–26). ROR and PRR, both frequentist methods, offer satisfactory sensitivity. ROR is effective at correcting for reporting biases in cases of low event counts, while PRR is less affected by under-reporting of AEs (24, 27). Nonetheless, their reliability may diminish when AE report numbers are limited (28). Bayesian algorithms, including BCPNN and MGPS, offer distinct advantages in terms of specificity, signal consistency, and minimization of false-positive rate. BCPNN enables efficient integration of data from diverse sources and supports cross-validation, whereas MGPS is particularly adept at detecting rare AEs (25, 29). To ensure accurate signal detection, we applied all four algorithms concurrently and only considered an AE signal positive if it satisfied the predefined thresholds across all methods. Positive AE signals at the PT level were defined as positive PTs; otherwise, they were considered negative. All four algorithms are based on 2 × 2 contingency tables, as shown in **Supplementary Table 1**. The formulas used and the conditions for signal generation are presented in **Supplementary Table 2**. Bonferroni correction was used to adjust for multiple comparisons and control type I error risk (30).

### 2.3 Time to onset analysis

Time to onset (TTO, defined as the duration from START_DT [date of medication initiation] to EVENT_DT [date of AE onset]) was summarised using the median, minimum, maximum, and interquartile ranges (IQR) (31). The cumulative incidence of AEs was estimated using the Kaplan-Meier method, and differences between groups were assessed by the log-rank test (32). Reports with missing or erroneous data were excluded from the analysis. The temporal patterns of TTO data were modeled with Weibull distribution modeling, where the shape parameter (β) defined three scenarios: β <1 with an upper limit of 95% confidence interval (CI) <1 indicates a decreasing risk over time (early failure), β ≈1 with a 95% CI of β included 1 indicated a constant risk (random failure), and β >1 with a lower limit of 95% CI  >1 signifies an increasing risk over time (wear-out failure) (33, 34).

### 2.4 Regression and statistical analysis

FAERS reports data containing patient information (sex, age, PTs, and TTO) were extracted, and those with missing data or fewer than one case of a positive PT were excluded from the analysis. Suspected variables were subjected to least absolute shrinkage and selection operator (LASSO) regression, and significant factors were subsequently incorporated into Cox proportional hazards models to determine independent risk factors of drug-related mortality (35). The starting point within the Cox regression is defined as the date of medication initiation (START_DT), and the endpoint is the date of fatal adverse event onset (EVENT_DT). Risk scores were calculated as the sum of each variable multiplied by its corresponding Cox regression coefficient, and patients were stratified into high- and low-risk groups based on the median value. Time-dependent receiver operating characteristic (ROC) curves were constructed to assess the diagnostic accuracy of the drug-related death risk prediction scores. A two-sided p<0.05 was considered statistically significant for all analyses. Data processing and statistical analyses were conducted using Microsoft Excel 2019 and R software (version 4.4.3).

## 3 Results

### 3.1 Descriptive characteristics

From Q4–2017 to Q4 2024, a total of 12,478,450 AE reports were obtained from the DEMO dataset. After removing duplicate entries, 715 reports were identified as being potentially associated with durvalumab plus tremelimumab treatment. Sex was documented in only 59.2% of cases (n=423), with males comprising the majority (n=348, 48.7%) and females accounting for 10.5% (n=75). Age was available in 64.1% of reports, with the highest proportion (n=244, 34.1%) falling within the 65-79-year age group. Most reports were submitted by physicians (n=656, 91.7%). Serious outcomes were reported in a substantial proportion of cases, including death (26.4%), disability (0.1%), hospitalization (27.0%), life-threatening events (10.5%), and other serious outcomes (24.6%). In terms of geographic distribution, Japan contributed the largest number of reports (71.3%), followed by the United States (9.1%) and France (5.3%). AE reports showed an increasing trend over time, with proportions of 7.3% in 2020, 23.5% in 2023, and 68.5% in 2024. The demographic characteristics of AE reports are summarized in **Table 1**.

**Table 1:** Demographic and clinical characteristics of durvalumab plus tremelimumab related AE reports in the FAERS database.

Parameters	Number of reports (%)
Sex	
Female	75 (10.5)
Male	348 (48.7)
Missing	292 (40.8)
Age	
<18	15 (2.1)
18-64	96 (13.4)
65-79	244 (34.1)
≥80	103 (14.4)
Missing	257 (35.9)
Occupation reporter	
Physician	656 (91.7)
Pharmacist	17 (2.4)
Health-professional	23 (3.2)
Consumer	7 (1.0)
Missing	12 (1.7)
Serious outcome	
Death	189 (26.4)
Disability	1 (0.1)
Hospitalization	193 (27.0)
Life-threatening events	75 (10.5)
Other serious outcomes	176 (24.6)
Missing	81 (11.3)
Reporting country	
Japan	510 (71.3)
United States	65 (9.1)
France	38 (5.3)
China	12 (1.7)
Canada	11 (1.5)
Other countries	79 (11.0)
Reporting year	
2020	52 (7.3)
2021	2 (0.3)
2022	3 (0.4)
2023	168 (23.5)
2024	490 (68.5)

AE, adverse event; FAERS, food and drug administration adverse event reporting system.

### 3.2 Safety signal detection results

**Figure 2:**
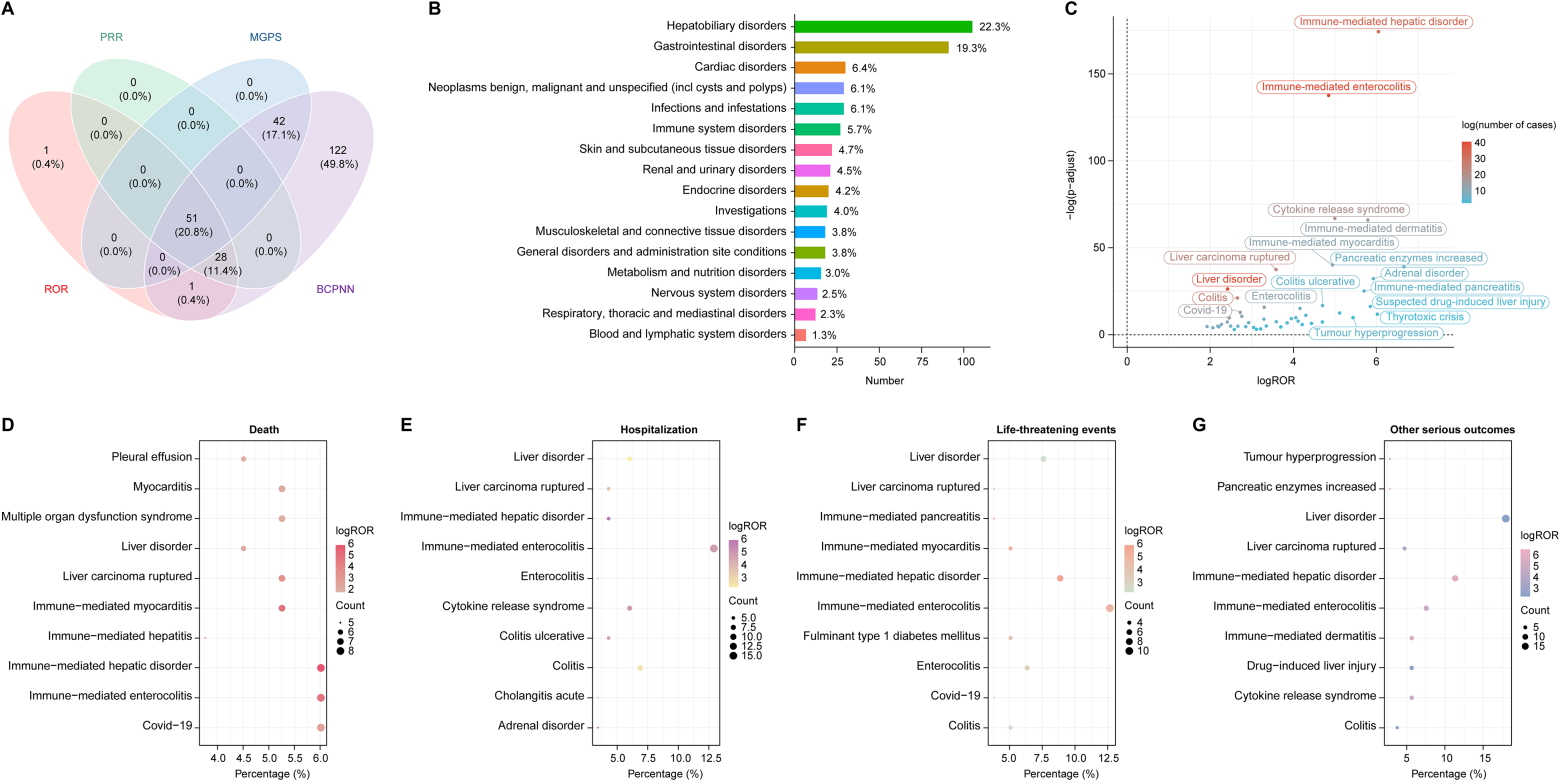
Disproportionality analysis and AEs distribution across SOC in HCC treated with durvalumab plus tremelimumab. **(A)** Venn diagram illustrating the overlap of positive AE signals identified by four algorithms: Reporting Odds Ratio (ROR), Proportional Reporting Ratio (PRR), Bayesian Confidence Propagation Neural Network (BCPNN), and Multi-Item Gamma Poisson Shrinker (MGPS); **(B)** Distribution of AEs categorized by SOC. Percentages were calculated as the number of cases within each SOC divided by the total 472 cases associated with the 51 PTs identified across 16 SOCs by all four algorithms; **(C)** Volcano plot displaying signal strength of PTs based on log ROR and -log10 (*p* value), with dot color representing the number of AE cases. P values were adjusted with Bonferroni test; **(D–G)** Distribution of top PTs associated with four clinical outcomes: **(D)** death, **(E)** hospitalization, **(F)** life-threatening events, and **(G)** other serious outcomes. The size and color of each dot represent case count and log ROR, respectively. AE, adverse event; SOC, system organ class; PT, preferred term; HCC, hepatocellular carcinoma.

The four algorithms combined identified a total of 51 positive PTs across 16 SOCs in 472 cases, as detailed in **Figure 2A** and **Supplementary Table 3**. The most frequently reported PTs included immune-mediated enterocolitis (n=41), liver disorder (n=40), immune-mediated hepatic disorder (n=33), colitis (n=26), and liver carcinoma ruptured (n=23) (**Table 2**). At the SOC level, the most commonly reported classifications were hepatobiliary disorders (105/472, 22.3%), gastrointestinal disorders (91/472, 19.3%), and cardiac disorders (30/472, 6.4%) (**Figure 2B**).

**Table 2:** The top 20 signals in the FAERS database were ranked by case count across both PT and SOC levels.

PT	SOC	Case (n)	ROR (95% CI)	PRR (95% CI)	χ2	IC (IC025)	EBGM (EBGM05)
Immune-mediated enterocolitis	Gastrointestinal disorders	41	28.82 (19.39-42.84)	28.1 (27.71-28.49)	649.99	4.12 (3.59)	17.41 (12.5)
Liver disorder	Hepatobiliary disorders	40	5.34 (3.83-7.45)	5.23 (4.91-5.56)	122.81	2.26 (1.78)	4.78 (3.62)
Immune-mediated hepatic disorder	Hepatobiliary disorders	33	66.12 (38.46-113.67)	64.76 (64.22-65.29)	829.26	4.73 (4.1)	26.5 (16.84)
Colitis	Gastrointestinal disorders	26	6.29 (4.16-9.52)	6.2 (5.79-6.61)	99.48	2.47 (1.88)	5.55 (3.92)
Liver carcinoma ruptured	Neoplasms benign, malignant and unspecified (incl cysts and polyps)	23	11.98 (7.53-19.05)	11.82 (11.36-12.28)	179.13	3.25 (2.59)	9.49 (6.44)
Cytokine release syndrome	Immune system disorders	19	31.92 (17.63-57.8)	31.55 (30.96-32.14)	324.94	4.22 (3.45)	18.65 (11.35)
Covid-19	Infections and infestations	15	6.59 (3.82-11.38)	6.54 (6-7.08)	61.25	2.54 (1.77)	5.81 (3.68)
Immune-mediated dermatitis	Skin and subcutaneous tissue disorders	14	55.43 (25.12-122.29)	54.95 (54.16-55.73)	326.38	4.63 (3.69)	24.74 (12.76)
Myocarditis	Cardiac disorders	14	5.48 (3.14-9.58)	5.45 (4.89-6)	45.19	2.31 (1.52)	4.95 (3.1)
Immune-mediated myocarditis	Cardiac disorders	12	30.7 (14.64-64.39)	30.47 (29.74-31.21)	200.62	4.19 (3.23)	18.28 (9.83)
Enterocolitis	Gastrointestinal disorders	12	9.84 (5.25-18.45)	9.77 (9.15-10.4)	77.15	3.03 (2.15)	8.15 (4.82)
Drug-induced liver injury	Hepatobiliary disorders	12	6.77 (3.68-12.47)	6.73 (6.12-7.33)	50.69	2.57 (1.72)	5.96 (3.57)
Renal disorder	Renal and urinary disorders	11	5.31 (2.83-9.94)	5.28 (4.65-5.9)	34.02	2.27 (1.38)	4.81 (2.84)
Pleural effusion	Respiratory, thoracic and mediastinal disorders	11	3.79 (2.04-7.03)	3.77 (3.15-4.38)	20.62	1.83 (0.96)	3.55 (2.11)
Myositis	Musculoskeletal and connective tissue disorders	10	4.82 (2.5-9.28)	4.8 (4.15-5.45)	27.09	2.14 (1.22)	4.42 (2.55)
Multiple organ dysfunction syndrome	General disorders and administration site conditions	9	4.54 (2.28-9.03)	4.52 (3.83-5.2)	22.35	2.07 (1.1)	4.18 (2.35)
Skin disorder	Skin and subcutaneous tissue disorders	8	4.62 (2.23-9.6)	4.6 (3.88-5.33)	20.43	2.09 (1.08)	4.26 (2.31)
Immune-mediated hepatitis	Hepatobiliary disorders	8	4.18 (2.02-8.64)	4.16 (3.44-4.89)	17.55	1.96 (0.95)	3.88 (2.11)
Pancreatic enzymes increased	Investigations	7	101.18 (26.14-391.64)	100.73 (99.38-102.09)	207.39	4.95 (3.61)	30.92 (9.96)
Adrenal disorder	Endocrine disorders	7	60.71 (19.25-191.48)	60.44 (59.29-61.59)	170.53	4.69 (3.39)	25.77 (9.85)

SOC, system organ class; PTs, preferred terms; ROR, reporting odds ratio; CI, confidence interval; PRR, proportional reporting ratio; IC, information component; IC025, the lower limit of 95% CI of the IC; EBGM, empirical Bayesian geometric mean; EBGM05, the lower limit of 95% CI of EBGM.

**Figure 3:**
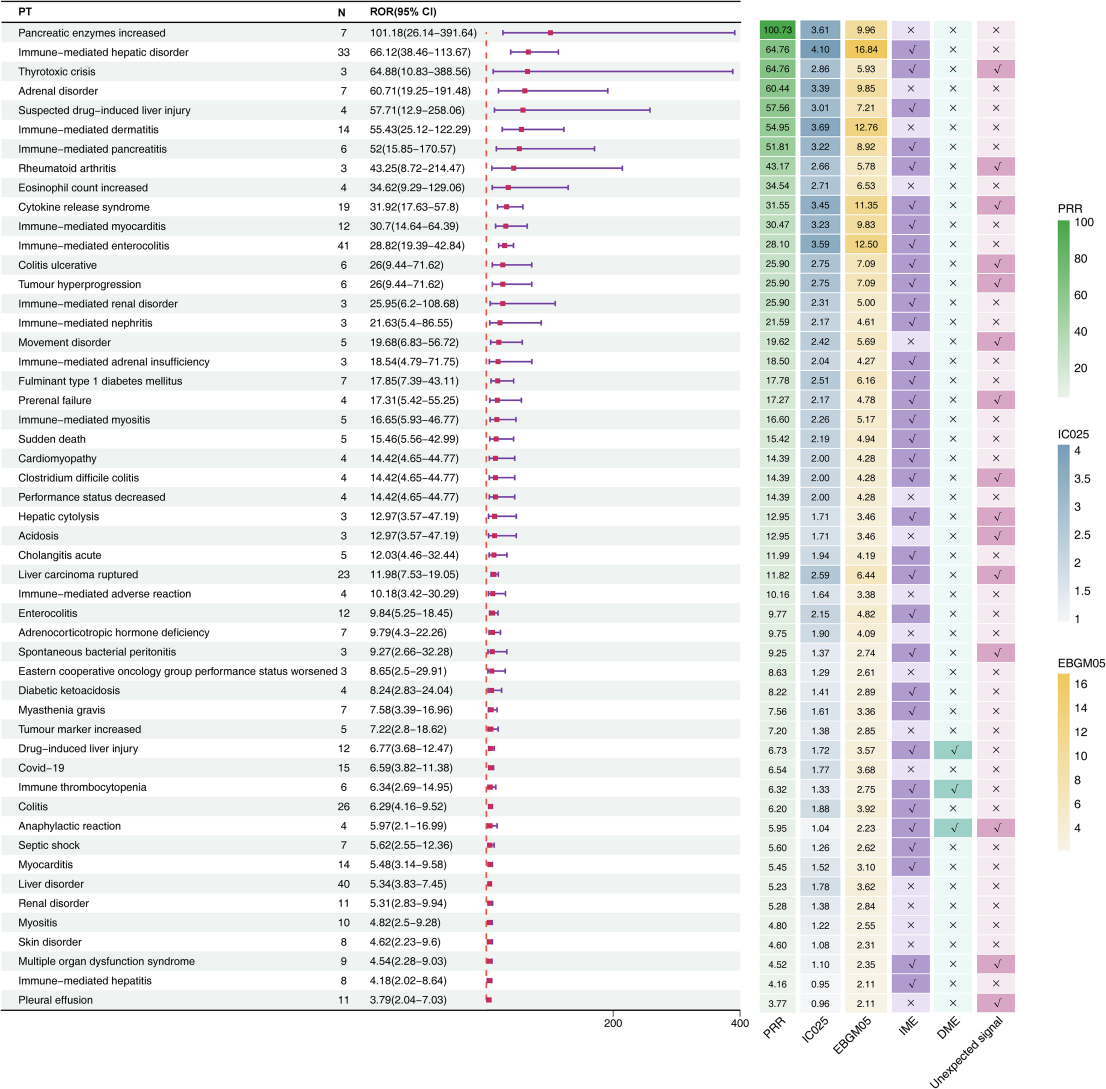
Signal detection at the PT level for durvalumab plus tremelimumab. The forest plot presents the top 20 preferred terms (PTs), ranked in descending order of reporting odds ratio (ROR), along with corresponding 95% confidence intervals, based on data from the FAERS database. The adjacent heatmap visualizes additional signal detection metrics—including PRR, IC025, EBGM05—as well as classification as unexpected signals, Important Medical Events (IMEs), or Designated Medical Events (DMEs). CI, confidence interval; ROR, Reporting Odds Ratio; PRR, Proportional Reporting Ratio; EBGM05, the lower limit of the 95% CI of EBGM; IC025, the lower limit of the 95% CI of the IC; SOC, system organ class; PT, preferred term; FAERS, FDA Adverse Event Reporting System.

Generally, higher ROR values indicated stronger associations with durvalumab plus tremelimumab. We specifically analyzed the top 20 PTs with the highest reporting ROR. As illustrated in **Figure 3**, pancreatic enzymes increased happened in 7 cases, with a highest ROR of 101.18 (95% CI, 26.14-391.64), indicating a strong signal for this particular AE. Other notable PTs included immune-mediated hepatic disorder (n=33; ROR, 66.12 [95% CI, 38.46-113.67]), immune-mediated enterocolitis (n=41; ROR, 28.82 [95% CI, 19.39-42.84]), cytokine release syndrome (n=19; ROR, 31.92 [95% CI, 17.63-57.8]), immune-mediated dermatitis (n=14; ROR, 55.43 [95% CI, 25.12-122.29]), and immune-mediated myocarditis (n=12; ROR, 30.7 [95% CI, 14.64-64.39]). After Bonferroni correction for multiple testing, these associations remained highly significant, confirming the robustness of the detected safety signals (**Figure 2C**). Further analysis revealed that immune-mediated hepatic disorder, immune-mediated enterocolitis, and liver disorder consistently ranked among the top ten PTs across four critical clinical outcomes: death, hospitalization, life-threatening events, and other serious outcomes (**Figures 2D–G**).

Six unexpected AEs within the top 20 signals ranked by ROR also appeared on the EMA’s IME list (**Figure 3**): thyrotoxic crisis (n=3, ROR: 64.88 [95% CI, 10.83-388.56], PRR: 64.76, EBGM05: 5.93, IC025: 2.86), rheumatoid arthritis (n=3, ROR: 43.25 [95% CI, 8.72-214.47], PRR: 43.17, EBGM05: 5.78, IC025: 2.66), cytokine release syndrome (n=19, ROR: 31.92 [95% CI, 17.63-57.8], PRR: 31.55, EBGM05: 11.35, IC025: 3.45), colitis ulcerative (n=6, ROR: 26 [95% CI, 9.44-71.62], PRR: 25.9, EBGM05: 7.09, IC025: 2.75), tumour hyperprogression (n=6, ROR: 26 [95% CI, 9.44-71.62], PRR: 25.9, EBGM05: 7.09, IC025: 2.75), and prerenal failure (n=4, ROR: 17.31 [95% CI, 5.42-55.25], PRR: 17.27, EBGM05: 4.78, IC025: 2.17).

Across all detected signals, drug-induced liver injury, immune thrombocytopenia, and anaphylactic reaction were classified simultaneously as DMEs and IMEs. Notably, anaphylactic reaction emerged as a novel signal not listed in the drug label.

### 3.3 Time to onset analysis

**Figure 4:**
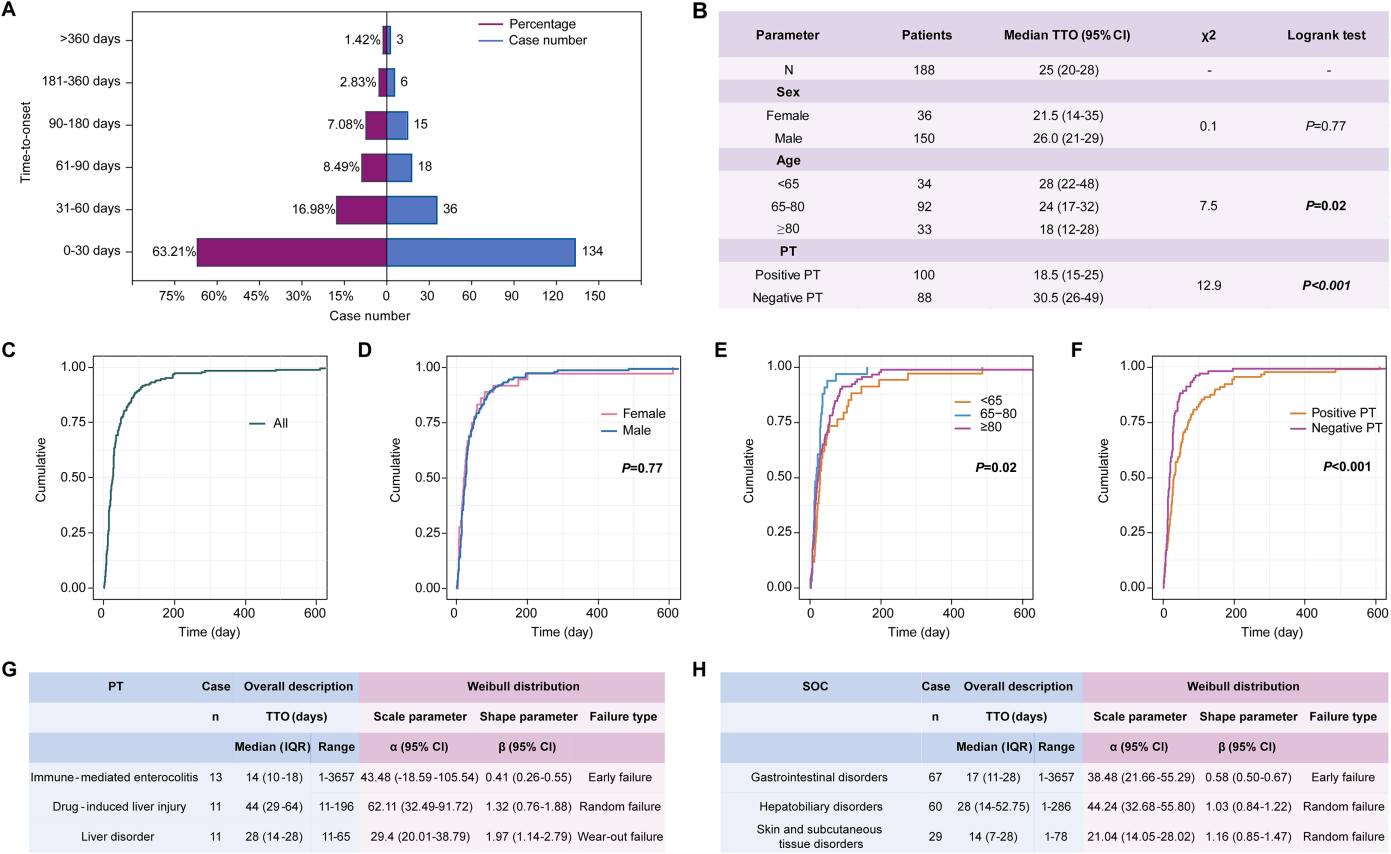
Time-to-onset (TTO) analysis in 188 patients with complete and precise information, encompassing a total of 212 reported AEs. **(A)** TTO of AEs related to durvalumab plus tremelimumab among the 212 AEs; **(B)** Log-rank test of TTO for 188 patients stratified by sex, age, and PTs; **(C)** Overall Kaplan-Meier curve showing the cumulative incidence of AE onset for 188 patients; **(D–F)** Kaplan-Meier curves stratified by sex **(C)**, age group **(D)**, and PT type **(E)** for 188 patients; **(G)** Weibull distribution of the top 3 PTs among the 188 patients; **(H)** Weibull distribution of the top 3 SOCs among the 188 patients. PT, preferred term; SOC, system organ class; TTO, time to onset; IQR, interquartile range; CI, confidence interval.

Complete and precise TTO information was available for 188 patients, encompassing a total of 212 reported AEs. The median TTO was 25 (95% CI, 20-28) days. The majority of AEs (134/212, 63.2%) occurred within the first 30 days after initiating durvalumab plus tremelimumab therapy, followed by a decline to 16.98% (36/212) between days 30-60, 8.49% (18/212) between days 60-90, and just 1.42% (3/212) beyond one year (**Figures 4A, C**).

To further determine potential factors influencing AE onset, the 188 patients were stratified by sex, age, and AE type (**Figure 4B**). As shown in **Figure 4D**, no significant difference in TTO was observed between females and males (*p* = 0.77), whereas age (*p* = 0.02, **Figure 4E**) and AE type (PT-positive vs. PT-negative) (*p* < 0.001, **Figure 4F**) were significantly associated with the time of onset.

Weibull distribution modeling was applied separately to characterize the temporal patterns of the top three most frequent PTs and SOCs among the 188 patients with complete TTO data. Immune-mediated enterocolitis (β, 0.41 [95% CI, 0.26-0.55]) demonstrated an early failure-type pattern, indicating a declining risk over time (**Figure 4G**). In contrast, drug-induced liver injury (β, 1.32 [95% CI, 0.76-1.88]) exhibited a random failure-type pattern, suggesting a constant risk. Liver disorder (β, 1.97 [95% CI, 1.14-2.79]) showed a wear-out failure-type pattern, implying increasing risk with prolonged treatment duration. At the SOC level, gastrointestinal disorders followed an early failure-type pattern, whereas hepatobiliary disorders and skin and subcutaneous tissue disorders were characterized by random failure-type distributions (**Figure 4H**).

### 3.4 Prognostic risk modeling

**Figure 5:**
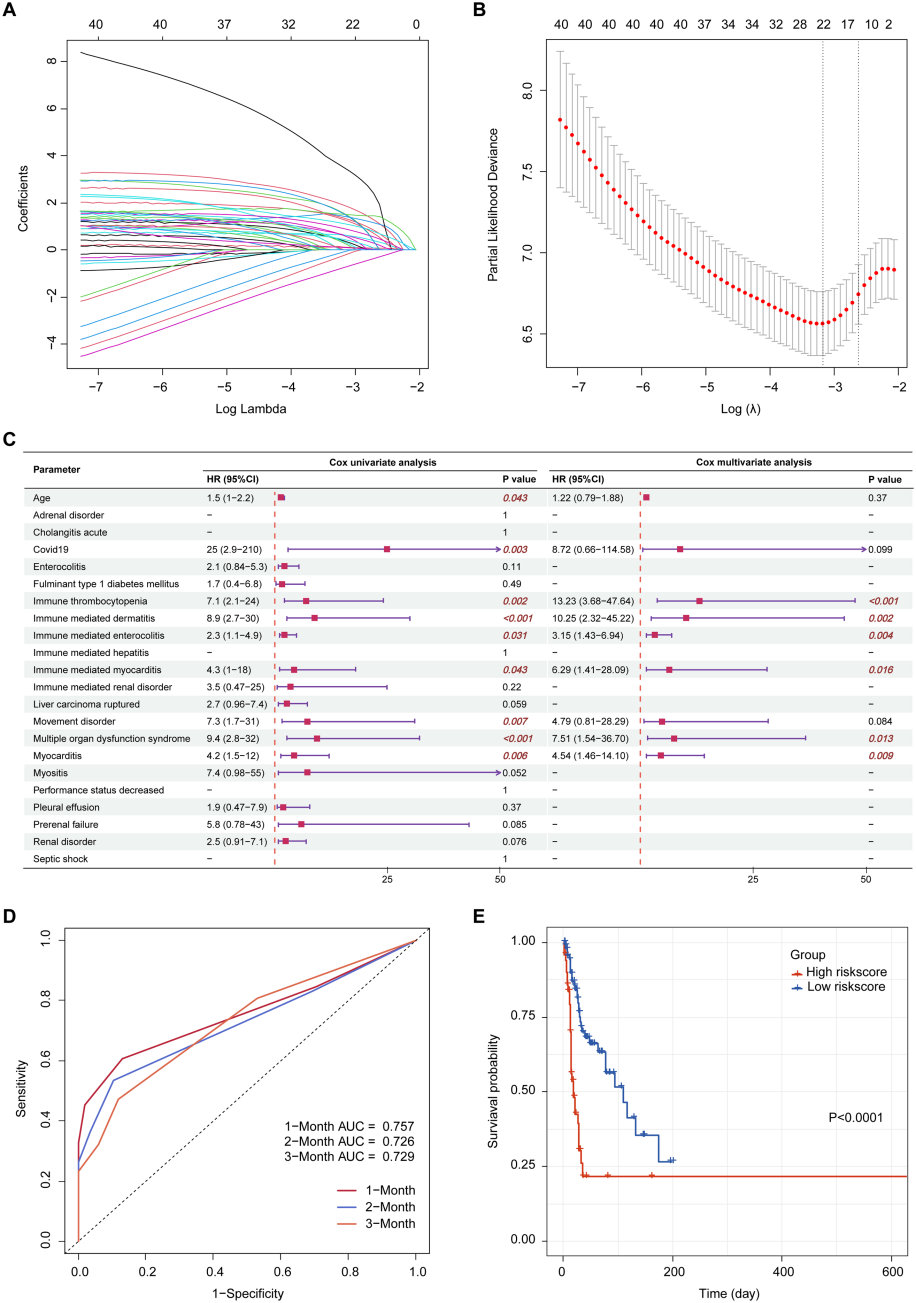
Prognostic risk modeling. **(A)** LASSO coefficient profiles of the suspected variables; **(B)** Optimal lambda selection based on minimum partial likelihood deviance; **(C)** Forest plot of univariate and multivariate Cox regression analyses; **(D)** Time-dependent ROC curves evaluating the predictive accuracy of the risk model at 1-, 2-, and 3-month intervals; **(E)** Kaplan-Meier survival curves comparing high- and low-risk groups stratified by median risk score. LASSO, least absolute shrinkage and selection operator; ROC, receiver operating characteristic; AUC, area under the curve; HR, harzard ratio; CI, confidence interval.

Suspected variables (including age and sex) were analyzed using LASSO regression on complete case data, yielding 22 variables for further analysis (**Figures 5A, B**). Multivariable Cox regression revealed six independent prognostic risk factors for drug-related mortality: immune thrombocytopenia, immune-mediated dermatitis, immune-mediated enterocolitis, immune-mediated myocarditis, multiple organ dysfunction syndrome, and myocarditis (**Figure 5C**). Based on the regression coefficients, the following risk score formula was established: Risk score = 2.5828 × immune thrombocytopenia + 2.3273 × immune-mediated dermatitis + 1.1487 × immune-mediated enterocolitis + 1.8384 × immune-mediated myocarditis + 2.0168 × multiple organ dysfunction syndrome + 1.5122 × myocarditis.

Patients were categorized into high- and low-risk groups according to the median risk score. Time-dependent ROC analysis showed the capacity of the risk score to predict drug-related mortality, with AUC values of 0.757, 0.726, and 0.729 at 1, 2, and 3 months, respectively (**Figure 5D**). Kaplan-Meier analysis revealed significantly poorer survival in the high-risk group (*p* < 0.0001, **Figure 5E**).

## 4 Disscussion

Durvalumab plus tremelimumab has shown promising results as a first-line regimen for patients with unresectable HCC. With over 5 years of follow-up, the phase III HIMALAYA trial recently demonstrated long-term survival benefits with 5-year overall survival rates of 28.7% and 50.7% in patients with disease control and significant tumour shrinkage, respectively (36). However, real-world data suggest that this combination should be used with caution in elderly patients or those with impaired liver function due to elevated toxicity risks (37). In light of these concerns, and given that current AE data are limited to monotherapies (38, 39), we conducted a pharmacovigilance analysis to characterize the safety profile observed in durvalumab plus tremelimumab based on FAERS pharmacovigilance data.

Analysis of baseline characteristics revealed that the reports were predominantly concentrated in male patients (48.7%) compared with females (10.5%), which may be attributed to the higher prevalence of HCC in males (40). Among reports with documented age, most patients were aged 65 years or older, suggesting that elderly patients may be more susceptible to treatment-related toxicities. The predominance of reports from Japan may reflect the real regional usage patterns and the extent of pharmacovigilance activities. Therefore, these findings should be interpreted with caution due to the substantial proportion of missing sex and age data, and imbalance in geographic distribution.

Among the 715 reports, the number of serious outcomes was 634 (88.7%), including death (26.4%), disability (0.1%), hospitalization (27.0%), life-threatening events (10.5%), and other serious outcomes (24.6%), underscoring the considerable risk profile of this combination therapy in real-world settings. With the expanding clinical use of durvalumab plus tremelimumab, these results highlight the importance of heightened vigilance and proactive monitoring, particularly in elderly patients, to minimize the risk of severe or fatal toxicities.

Our study identified several notable PTs with strong disproportionality signals, including immune-mediated hepatic disorder, immune-mediated enterocolitis, and cytokine release syndrome, which differ from those reported in a previous FEARS study of durvalumab plus tremelimumab, such as death, malignant neoplasm progression, and diarrhea (41). Such variation is expected and may reflect differences in study focus and analytical methods. At the SOC level, hepatobiliary, gastrointestinal, and cardiac disorders were the most frequently reported in our study, consistent with the known toxicity profile of ICIs (42). However, the potential associations derived from spontaneous reporting data alone should be interpreted with caution and validated in future studies.

Of the 51 positive PTs, over 25% of which were immune-mediated, suggesting a notable risk of immune-related side effects for this dual-ICI therapy. Immune-mediated enterocolitis, hepatic disorder, myocarditis, and dermatitis were the most frequently reported immune-related events, consistent with the toxicity profile observed in the HIMALAYA trial (11). Compared with the commonly used ipilimumab-nivolumab regimen, the overall spectrum of immune-related events was broadly similar. However, among the 51 PTs analyzed, immune-mediated optic neuritis and cystitis (the most prominent AEs with ipilimumab-nivolumab) were not observed, possibly reflecting distinct immunomodulatory mechanisms between the two combination therapies (43).

Based on prior studies, the immune-mediated enterocolitis can progress to life-threatening complications such as colonic ulceration, perforation, and peritonitis (44–46), underscoring the importance of early recognition of symptoms like diarrhea and abdominal pain and timely use of corticosteroids or biologics. Immune-mediated hepatic disorders are also frequently observed in patients with HCC, largely due to the tumour’s location, pre-existing hepatic impairment (47). However, distinguishing true drug-related toxicity from underlying disease progression or aggressive tumor biology in FAERS remains a major challenge. Although infrequent, cardiotoxicity such as lethal myocarditis accompanied by myositis can be fatal (48, 49), necessitating routine cardiac monitoring. While generally manageable, the incidence of grade ≥3 skin toxicity increases significantly with dual ICI therapy (approximately 4%) from monotherapy (<1%) (50, 51), and these events are strongly correlated with treatment response (52), warranting careful management. In summary, immune-mediated toxicities are widely recognized as major contributors to hospitalization and fatal outcomes during ICI therapy, as also demonstrated in our study. Their inclusion in the IME list lends further support to the reliability of our findings to a certain extent. In this context, patients with pre-existing liver disease, autoimmune disorders, or cardiovascular comorbidities should be closely monitored.

Beyond the expected spectrum of toxicities, several signals listed in the IME but not included in the product label, including cytokine release syndrome, multiple organ dysfunction syndrome, thyrotoxic crisis, and anaphylactic reaction, were also identified (53, 54). Although multiple organ dysfunction syndrome has not previously been reported as a positive safety signal in HCC patients receiving combination immunotherapy, it may result from systemic autoimmune or inflammatory responses triggered by ICIs (55). Additionally, patients with HCC secondary to chronic liver disease are inherently prone to multi-organ dysfunction, which may overlap with or exacerbate irAEs, warranting heightened clinical vigilance in this population (56). Anaphylactic reaction deserves close attention, given its inclusion in the EMA’s IME and DME lists and the limited feasibility of excluding hypersensitive patients in real-world practice. These findings offer new insights into drug safety surveillance, underscoring the importance of awareness for these rare but severe AEs.

In this study, the median TTO was 25 days, shorter than the 41 days reported for durvalumab monotherapy (3). This difference may result from more rapid immunologic effects caused by the synergistic immune activation of dual ICIs, highlighting the need for vigilant monitoring during the initial treatment phase. Baseline assessment of immune status, close follow-up within the first 1–3 months, rapid intervention protocols, and patient education to ensure timely reporting are key strategies that collectively help reduce risk and improve treatment adherence and outcomes. Stratified analyses showed that age and AE type significantly influenced TTO. Weibull modeling revealed distinct temporal patterns for the three most frequently reported PTs: immune-mediated enterocolitis showed a decreasing risk over time, drug-induced liver injury maintained a constant risk, while liver disorder demonstrated a rising risk with prolonged treatment duration. Despite being preliminary, these results offer clinicians important guidance to anticipate AE onset timing and customize monitoring schedules accordingly.

By integrating LASSO and Cox regression analyses, we identified six independent risk factors potentially associated with drug-related mortality: immune-mediated enterocolitis, immune-mediated dermatitis, immune-mediated myocarditis, immune thrombocytopenia, myocarditis, and multiple organ dysfunction syndrome. A risk score model was exploratorily constructed based on these factors, with time-dependent ROC analysis showing acceptable capacity for drug-related mortality risk prediction. However, this risk score model should be interpreted with caution due to the inherent constraints of spontaneous reporting data (which present challenges to establishing precise indices like Common Terminology Criteria for Adverse Events grading) and a lack of external validation, requiring its further refinement by integrating additional clinical indicators and relevant information.

This study has several inherent limitations. First, as a global spontaneous reporting system, the FAERS database collects submissions from healthcare professionals, consumers, and pharmaceutical companies, which introduces inherent selection biases, including variation in the ethnicity and geographic origin of cases. Additionally, limited data availability precluded regional stratified sensitivity analyses, which warrant further investigation as the database grows. Moreover, potential biases may arise from duplicate or censored reports, as well as incomplete patient-level information (lack of clinical course, baseline liver function, and complete medication records), which are critical for accurately assessing adverse events (57, 58). Furthermore, while disproportionality analysis identified safety signals associated with durvalumab plus tremelimumab in HCC patients, the potential causal relationships observed between these AEs and the drug combination require further validation in future prospective controlled studies.

## 5 Conclusion

Overall, this study characterizes the adverse event profile of durvalumab plus tremelimumab in patients with HCC using the FAERS database, providing key pharmacological insights. Nevertheless, these findings require confirmation in future prospective research.

## Data Availability

The original contributions presented in the study are included in the article/**Supplementary Material**. Further inquiries can be directed to the corresponding author.
