# Supplementary material for: A real-world drug safety surveillance study from the FAERS database of hepatocellular carcinoma patients receiving durvalumab in combination with tremelimumab
Source: Front Immunol. 2025 Oct 29;16:1657398. doi: 10.3389/fimmu.2025.1657398 (PMC12605205; doi:10.3389/fimmu.2025.1657398)
Supplement: Supplementary file 1 [file Table1.docx]

**Supplementary Material**

**Supplementary Table 1. Fourfold table for calculation, used for comparing the association between a specific drug and the occurrence of a specific adverse event.**

|  | **Target adverse reaction** | **Other adverse reactions** | **Total** |
| --- | --- | --- | --- |
| Durvalumab plus Tremelimumab | a | b | a+b |
| Non-[combination therapy](https://zhida.zhihu.com/search?content_id=186695474&content_type=Article&match_order=1&q=combination+therapy&zhida_source=entity) | c | d | c+d |
| Total | a+c | b+d | N=a+b+c+d |

**Supplementary Table 2. Four main algorithms are used to evaluate the correlation between Durvalumab plus Tremelimumab and AEs. This includes ROR, PRR, BCPNN and MGPS methods with formulas and thresholds.**

| **Method** | **Formula** | **Threshold** |
| --- | --- | --- |
| ROR | $ROR=\frac{\left( a/b \right)}{\left( c/d \right)}=\frac{ad}{bc}$ | a ≥ 3 and 95% CI (lower limit) > 1 |
|  | $SE\left( lnROR \right)=\sqrt{\left( \frac{1}{a}+\frac{1}{b}+\frac{1}{c}+\frac{1}{d} \right)}$ |  |
|  | $95\%CI=e^{\ln\left( ROR \right)\pm1.96\sqrt{\left( \frac{1}{a}+\frac{1}{b}+\frac{1}{c}+\frac{1}{d} \right)}}$ |  |
| PRR | $PRR=\frac{a/{a+b}}{c/{c+d}}$ | a>3, PRR>2 and χ2 > 4 |
|  | $SE\left( lnPRR \right)=\sqrt{\left( \frac{1}{a}-\frac{1}{a+b}+\frac{1}{c}-\frac{1}{c+d} \right)}$ |  |
|  | $95\%CI=e^{\ln\left( PRR \right)\pm1.96\sqrt{\left( \frac{1}{a}-\frac{1}{a+b}+\frac{1}{c}-\frac{1}{c+d} \right)}}$ |  |
|  | $x^{2}=\frac{\left( \left\vert ab-cd \right\vert-N/2 \right)^{2}\times N}{\left( a+b \right)\left( c+d \right)\left( a+c \right)\left( b+d \right)}$ |  |
| BCPNN | $IC=Log2\frac{p\left( x,y \right)}{p\left( x \right)p\left( y \right)}=Log2\frac{a\left( a+b+c+d \right)}{\left( a+b \right)\left( a+c \right)}$ | IC025 > 0 |
|  | $E\left( IC \right)=Log2\frac{\left( a+\gamma11 \right)\left( a+b+c+d+\alpha\right)\left( a+b+c+d+\beta\right)}{\left( a+b+c+d+\gamma\right)\left( a+b+\alpha1 \right)\left( a+c+\beta1 \right)}$ |  |
|  | $V\left( IC \right)=\frac{1}{{(ln2)}^{2}}\left\{ \left[ \frac{\left( a+b+c+d \right)-a+\gamma-\gamma11}{\left( a+\gamma11 \right)\left( 1+a+b+c+d+\gamma\right)} \right]+\left[ \frac{\left( a+b+c+d \right)-\left( a+b \right)+\alpha-\alpha1}{\left( a+b+\alpha1 \right)\left( 1+a+b+c+d+\alpha\right)} \right]+\left[ \frac{\left( a+b+c+d \right)-\left( a+c \right)+\beta-\beta1}{\left( a+c+\beta1 \right)\left( 1+a+b+c+d+\beta\right)} \right] \right\}$ |  |
|  | $\gamma=\gamma11\frac{\left( a+b+c+d+\alpha\right)\left( a+b+c+d+\beta\right)}{\left( a+b+\alpha1 \right)\left( a+c+\beta1 \right)}$ |  |
|  | $IC-2SD=E\left( IC \right)-2\sqrt{V\left( IC \right)}$ |  |
| MGPS | $EBGM=\frac{a/{a+b+c+d}}{\left( a+c \right)\left( a+b \right)}$ | EBGM05 > 2 |
|  | $95\%CI=e^{\ln\left( EBGM \right)\pm1.96\sqrt{\left( \frac{1}{a}+\frac{1}{b}+\frac{1}{c}+\frac{1}{d} \right)}}$ |  |

**Supplementary Table 3. All signals associated with Durvalumab plus Tremelimumab at the PTs and SOC levels in the FAERS database were ranked by case count across both PT and SOC levels.**

| **PT** | **SOC** | Case  (n) | **ROR**  **(95%CI)** | **PRR**  **(95% CI)** | **χ2** | **IC**  **(IC025)** | **EBGM**  **(EBGM05)** |
| --- | --- | --- | --- | --- | --- | --- | --- |
| Immune-mediated enterocolitis | Gastrointestinal disorders | 41 | 28.82(19.39-  42.84) | 28.1(27.71-  28.49) | 649.99 | 4.12(3.59) | 17.41(12.5) |
| Liver disorder | Hepatobiliary disorders | 40 | 5.34(3.83-  7.45) | 5.23(4.91-  5.56) | 122.81 | 2.26(1.78) | 4.78(3.62) |
| Immune-mediated hepatic disorder | Hepatobiliary disorders | 33 | 66.12(38.46-  113.67) | 64.76(64.22-  65.29) | 829.26 | 4.73(4.1) | 26.5(16.84) |
| Colitis | Gastrointestinal disorders | 26 | 6.29(4.16-  9.52) | 6.2(5.79-  6.61) | 99.48 | 2.47(1.88) | 5.55(3.92) |
| Liver carcinoma ruptured | Neoplasms benign, malignant and unspecified (incl cysts and polyps) | 23 | 11.98(7.53-  19.05) | 11.82(11.36-  12.28) | 179.13 | 3.25(2.59) | 9.49(6.44) |
| Cytokine release syndrome | Immune system disorders | 19 | 31.92(17.63-  57.8) | 31.55(30.96-  32.14) | 324.94 | 4.22(3.45) | 18.65(11.35) |
| Covid-19 | Infections and infestations | 15 | 6.59(3.82-  11.38) | 6.54(6-  7.08) | 61.25 | 2.54(1.77) | 5.81(3.68) |
| Immune-mediated dermatitis | Skin and subcutaneous tissue disorders | 14 | 55.43(25.12-  122.29) | 54.95(54.16-  55.73) | 326.38 | 4.63(3.69) | 24.74(12.76) |
| Myocarditis | Cardiac disorders | 14 | 5.48(3.14-  9.58) | 5.45(4.89-  6) | 45.19 | 2.31(1.52) | 4.95(3.1) |
| Immune-mediated myocarditis | Cardiac disorders | 12 | 30.7(14.64-  64.39) | 30.47(29.74-  31.21) | 200.62 | 4.19(3.23) | 18.28(9.83) |
| Enterocolitis | Gastrointestinal disorders | 12 | 9.84(5.25-  18.45) | 9.77(9.15-  10.4) | 77.15 | 3.03(2.15) | 8.15(4.82) |
| Drug-induced liver injury | Hepatobiliary disorders | 12 | 6.77(3.68-  12.47) | 6.73(6.12-  7.33) | 50.69 | 2.57(1.72) | 5.96(3.57) |
| Renal disorder | Renal and urinary disorders | 11 | 5.31(2.83-  9.94) | 5.28(4.65-  5.9) | 34.02 | 2.27(1.38) | 4.81(2.84) |
| Pleural effusion | Respiratory, thoracic and mediastinal disorders | 11 | 3.79(2.04-  7.03) | 3.77(3.15-  4.38) | 20.62 | 1.83(0.96) | 3.55(2.11) |
| Myositis | Musculoskeletal and connective tissue disorders | 10 | 4.82(2.5-  9.28) | 4.8(4.15-  5.45) | 27.09 | 2.14(1.22) | 4.42(2.55) |
| Multiple organ dysfunction syndrome | General disorders and administration site conditions | 9 | 4.54(2.28-  9.03) | 4.52(3.83-  5.2) | 22.35 | 2.07(1.1) | 4.18(2.35) |
| Skin disorder | Skin and subcutaneous tissue disorders | 8 | 4.62(2.23-  9.6) | 4.6(3.88-  5.33) | 20.43 | 2.09(1.08) | 4.26(2.31) |
| Immune-mediated hepatitis | Hepatobiliary disorders | 8 | 4.18(2.02-  8.64) | 4.16(3.44-  4.89) | 17.55 | 1.96(0.95) | 3.88(2.11) |
| Pancreatic enzymes increased | Investigations | 7 | 101.18(26.14-  391.64) | 100.73(99.38-102.09) | 207.39 | 4.95(3.61) | 30.92(9.96) |
| Adrenal disorder | Endocrine disorders | 7 | 60.71(19.25-  191.48) | 60.44(59.29-  61.59) | 170.53 | 4.69(3.39) | 25.77(9.85) |
| Fulminant type 1 diabetes mellitus | Metabolism and nutrition disorders | 7 | 17.85(7.39-  43.11) | 17.78(16.9-  18.66) | 78.53 | 3.69(2.51) | 12.88(6.16) |
| Adrenocorticotropic hormone deficiency | Endocrine disorders | 7 | 9.79(4.3-  22.26) | 9.75(8.93-  10.57) | 44.86 | 3.02(1.9) | 8.14(4.09) |
| Myasthenia gravis | Nervous system disorders | 7 | 7.58(3.39-  16.96) | 7.56(6.75-  8.36) | 33.91 | 2.72(1.61) | 6.58(3.36) |
| Septic shock | Infections and infestations | 7 | 5.62(2.55-  12.36) | 5.6(4.81-  6.38) | 23.41 | 2.34(1.26) | 5.07(2.62) |
| Immune-mediated pancreatitis | Gastrointestinal disorders | 6 | 52(15.85-  170.57) | 51.81(50.62-  52.99) | 135.91 | 4.59(3.22) | 24.09(8.92) |
| Colitis ulcerative | Gastrointestinal disorders | 6 | 26(9.44-  71.62) | 25.9(24.89-  26.91) | 89.8 | 4.05(2.75) | 16.56(7.09) |
| Tumour hyperprogression | Neoplasms benign, malignant and unspecified (incl cysts and polyps) | 6 | 26(9.44-  71.62) | 25.9(24.89-  26.91) | 89.8 | 4.05(2.75) | 16.56(7.09) |
| Immune thrombocytopenia | Blood and lymphatic system disorders | 6 | 6.34(2.69-  14.95) | 6.32(5.46-  7.17) | 23.44 | 2.5(1.33) | 5.64(2.75) |
| Movement disorder | Nervous system disorders | 5 | 19.68(6.83-  56.72) | 19.62(18.57-  20.68) | 60.77 | 3.79(2.42) | 13.8(5.69) |
| Immune-mediated myositis | Musculoskeletal and connective tissue disorders | 5 | 16.65(5.93-  46.77) | 16.6(15.57-  17.63) | 52.97 | 3.62(2.26) | 12.27(5.17) |
| Sudden death | General disorders and administration site conditions | 5 | 15.46(5.56-  42.99) | 15.42(14.4-  16.44) | 49.69 | 3.54(2.19) | 11.62(4.94) |
| Cholangitis acute | Hepatobiliary disorders | 5 | 12.03(4.46-  32.44) | 11.99(11-  12.98) | 39.44 | 3.26(1.94) | 9.6(4.19) |
| Tumour marker increased | Investigations | 5 | 7.22(2.8-  18.62) | 7.2(6.25-8.14) | 22.87 | 2.66(1.38) | 6.31(2.85) |
| Suspected drug-induced liver injury | Hepatobiliary disorders | 4 | 57.71(12.9-  258.06) | 57.56(56.07-  59.06) | 95.29 | 4.66(3.01) | 25.24(7.21) |
| Eosinophil count increased | Investigations | 4 | 34.62(9.29-  129.06) | 34.54(33.22-  35.85) | 72.38 | 4.3(2.71) | 19.63(6.53) |
| Prerenal failure | Renal and urinary disorders | 4 | 17.31(5.42-  55.25) | 17.27(16.11-  18.43) | 43.8 | 3.66(2.17) | 12.62(4.78) |
| Cardiomyopathy | Cardiac disorders | 4 | 14.42(4.65-  44.77) | 14.39(13.26-  15.52) | 37.39 | 3.47(2) | 11.04(4.28) |
| Clostridium difficile colitis | Infections and infestations | 4 | 14.42(4.65-  44.77) | 14.39(13.26-  15.52) | 37.39 | 3.47(2) | 11.04(4.28) |
| Performance status decreased | General disorders and administration site conditions | 4 | 14.42(4.65-  44.77) | 14.39(13.26-  15.52) | 37.39 | 3.47(2) | 11.04(4.28) |
| Immune-mediated adverse reaction | Immune system disorders | 4 | 10.18(3.42-  30.29) | 10.16(9.07-  11.25) | 26.74 | 3.07(1.64) | 8.41(3.38) |
| Diabetic ketoacidosis | Metabolism and nutrition disorders | 4 | 8.24(2.83-  24.04) | 8.22(7.16-  9.29) | 21.33 | 2.82(1.41) | 7.07(2.89) |
| Anaphylactic reaction | Immune system disorders | 4 | 5.97(2.1-  16.99) | 5.95(4.91-  7) | 14.5 | 2.42(1.04) | 5.35(2.23) |
| Thyrotoxic crisis | Endocrine disorders | 3 | 64.88(10.83-  388.56) | 64.76(62.97-  66.55) | 75.33 | 4.73(2.86) | 26.5(5.93) |
| Rheumatoid arthritis | Musculoskeletal and connective tissue disorders | 3 | 43.25(8.72-  214.47) | 43.17(41.57-  44.77) | 61.8 | 4.47(2.66) | 22.09(5.78) |
| Immune-mediated renal disorder | Renal and urinary disorders | 3 | 25.95(6.2-  108.68) | 25.9(24.47-  27.33) | 44.9 | 4.05(2.31) | 16.56(5) |
| Immune-mediated nephritis | Renal and urinary disorders | 3 | 21.63(5.4-  86.55) | 21.59(20.2-  22.97) | 39.27 | 3.88(2.17) | 14.72(4.61) |
| Immune-mediated adrenal insufficiency | Endocrine disorders | 3 | 18.54(4.79-  71.75) | 18.5(17.15-  19.85) | 34.77 | 3.73(2.04) | 13.25(4.27) |
| Hepatic cytolysis | Hepatobiliary disorders | 3 | 12.97(3.57-  47.19) | 12.95(11.66-  14.24) | 25.46 | 3.35(1.71) | 10.19(3.46) |
| Acidosis | Metabolism and nutrition disorders | 3 | 12.97(3.57-  47.19) | 12.95(11.66-  14.24) | 25.46 | 3.35(1.71) | 10.19(3.46) |
| Spontaneous bacterial peritonitis | Infections and infestations | 3 | 9.27(2.66-  32.28) | 9.25(8.01-  10.5) | 18.19 | 2.96(1.37) | 7.8(2.74) |
| Eastern cooperative oncology group performance status worsened | Investigations | 3 | 8.65(2.5-  29.91) | 8.63(7.4-  9.87) | 16.88 | 2.88(1.29) | 7.36(2.61) |
